# Dietary Glucosamine: Effects and Mechanisms in Relation to Production Performance, Eggshell Quality, and Liver Health of Aged Laying Hens

**DOI:** 10.3390/ani16060910

**Published:** 2026-03-13

**Authors:** Wenting Gao, Yanan Wang, Ping Gong, Shahid Ali Rajput, Huanbin Wang, Shengqiang Ye, Yu Yang

**Affiliations:** 1 Institute of Animal Husbandry and Veterinary, Wuhan Academy of Agricultural Sciences, Wuhan 430208, China; gwt19940310@inha.edu (W.G.); gongping@wuhanagri.com (P.G.); yeshengqiang@wuhanagri.com (S.Y.); 2 Department of Biological Sciences and Bioengineering, Inha University, Incheon 22212, Republic of Korea; 3 Department of Animal and Dairy Sciences, Faculty of Veterinary and Animal Sciences, Muhammad Nawaz Shareef University of Agriculture, Multan 66000, Pakistan; shahid.ali@mnsuam.edu.pk; 4 Department of Animal Nutrition and Feed Science, College of Animal Science and Technology, Huazhong Agricultural University, Wuhan 430070, China; whbin@webmail.hzau.edu.cn

**Keywords:** glucosamine, elderly laying hens, eggshell quality, serum biochemical indices, serum antioxidant indices, hepatic steatosis, transcriptome sequencing, eggshell matrix protein

## Abstract

The aim of this study is to investigate the effects and mechanisms of GS in relation to the production performance, eggshell quality, and liver health of aged laying hens. A total of 144 elderly laying hens were randomly divided into a control group, a 0.15% GS group, and a 0.35% GS group and fed for 4 weeks. The experimental results revealed that GS can significantly improve the laying rate, eggshell strength, and eggshell thickness of aged laying hens and reduce the feed-to-egg ratio (*p* < 0.05). Specifically, 0.35% GS can alleviate symptoms of hepatic steatosis; significantly reduce the levels of AST, TG, and MDA in serum; and increase the levels of ALB and GSH-Px (*p* < 0.05). The sequencing results demonstrated that GS significantly upregulated the expression of fatty acid breakdown and antioxidant genes, such as *PPARA*, *ACOX1*, *GSTT1*, and *CAT*, in the liver, while downregulating the expression of fatty acid synthesis and inflammatory genes such as *PPARG*, *FASN*, *TGFBR2*, and *TNF-α* (*p* < 0.05). Meanwhile, GS also increased the expression of uterine eggshell matrix protein genes (*p* < 0.05). In summary, GS improves liver health by regulating fatty acid metabolism, inflammation, and antioxidant pathways and enhances eggshell quality by promoting eggshell matrix protein synthesis.

## Simple Summary

In response to common issues related to poor liver health and decreased eggshell quality in elderly laying hens, this study systematically evaluated the therapeutic effect of glucosamine (GS). The results indicate that GS can significantly improve the laying rate, eggshell quality, and liver health of elderly laying hens. The biological mechanism behind this is that GS upregulates the expression of fatty acid breakdown, antioxidant, and eggshell matrix protein genes and downregulates the expression of lipid synthesis and inflammatory genes in the liver. In summary, this study systematically elucidated the effects and molecular mechanisms of GS and explored its role in improving the production performance and liver health of elderly laying hens, providing potential nutritional strategies and a scientific basis to improve the liver health, eggshell quality, and breeding efficiency of elderly laying hens.

## 1. Introduction

As the productive lifespan of laying hens is extended to 72 weeks of age, two prevalent and interrelated challenges in late-stage production have been brought into sharp focus: compromised liver health and deteriorating eggshell quality [1]. Due to the continuous high-intensity egg laying cycle, elderly laying hens are prone to lipid metabolism disorders, inflammation, and oxidative stress damage in their liver, which can lead to decreased liver health, abnormal lipid deposition, and the obstruction of the 25-hydroxyvitamin D_3_ synthesis process [2]. Laying hens require approximately 4 g of calcium (equivalent to 10 g of CaCO_3_) daily, and insufficient synthesis of 25-hydroxyvitamin D_3_, a key regulator of calcium metabolism, directly impairs intestinal calcium absorption, thereby leading to osteoporosis and an inadequate supply of raw materials for eggshell calcification [2,3]. On the other hand, with age the function of reproductive organs, such as the uterus, in laying hens declines synchronously, and eggshell matrix synthesis and calcification processes are further hindered [4,5]. Under the combined action of the above dual mechanisms, the eggshell’s structural integrity and quality continue to decline, manifested by an increase in brittleness and a significant increase in breakage rates [1,2,3,4,5]. The pathological changes in the quality of the liver and eggshells have a slow development process, which is difficult to detect at early stages and is often discovered only when the problem accumulates to a significant stage, resulting in delayed intervention measures and ultimately causing significant and sustained economic losses [6]. Therefore, exploring nutritional intervention strategies that can effectively improve the liver health of aged laying hens, enhance antioxidant capacity, alleviate metabolic stress and regulate calcium metabolism is of great practical significance for improving eggshell quality, extending feeding cycles, and improving breeding efficiency.

GS is a naturally occurring amino monosaccharide that can be directly prepared through modern fermentation processes or extracted from the shells of crustaceans, such as shrimp and crabs, via chemical processes [7,8]. It benefits from broad sources and sufficient production and is currently widely used for the treatment of joint inflammation [7,8]. GS, as a precursor substance for the synthesis of glycosaminoglycans and proteoglycans in animal bodies, not only has various physiological effects—such as the regulation of immunity, antioxidation, and anti-inflammatory processes—but also plays a key regulatory role in eggshell quality during the formation process [8,9]. Research has shown that glycosaminoglycans synthesized from GS have a positive regulatory effect on eggshell quality, while proteoglycans synthesized from GS are crucial for regulating the nucleation sites, growth direction, and microstructure of calcium carbonate crystals in eggshells [9,10]. A study by Wang et al. (2024) determined that adding GS to the diet of laying hens during the initial period of production can increase the calcium content and strength of eggshells, thereby improving the production performance of laying hens [8]. Li et al. (2023) reported that GS can alleviate high-fat-diet-induced fatty liver disease in mice, improve antioxidant capacities, and alleviate liver inflammation [9]. Ryu et al. (2025) also determined that GS has an alleviating effect on human fatty liver disease [11]. Although the above studies provide evidence that GS may improve eggshell quality and liver metabolic diseases, the comprehensive effects of GS on the special population of elderly laying hens have not yet been reported.

To fill this research gap, this study focused on 390-day-old laying hens and investigated the effects of different doses of GS on their production performance, eggshell quality, liver health, serum biochemistry, and antioxidant indicators. Furthermore, by utilizing transcriptome sequencing and RT-qPCR technology, the molecular mechanism of GS in relation to improvements in liver health and eggshell quality was explored, providing an important theoretical basis for the development of new feed additives to enhance the production performance and health of elderly laying hens.

## 2. Materials and Methods

### 2.1. Experimental Animals, Diet, and Experimental Design

This experiment was reviewed and approved by the Animal Ethics Committee (Ethics Code: HZAUCH-2022-0011). A total of 144 elderly Jingfen No. 6 laying hens (Pink-shelled eggs) that were 390 days old were selected and randomly divided into three groups: the control group (fed with basic diet), 0.15% GS group (supplemented with 0.15% GS in the basic diet), and 0.35% GS group (supplemented with 0.35% GS in the basic diet). Each group had 8 replicates, with 6 hens per replicate. All laying hens were housed in an environmentally controlled, enclosed facility, where they were kept in stainless steel cages with ad libitum access to feed and water. The building’s windows remained sealed to maintain stable internal conditions, with relative humidity maintained at 60 ± 5%. The chicken coop possessed a constant LED lighting system (white light), with a light time of 16 h per day and a lighting intensity of 20 lux. The composition and nutritional level of the basic diet were formulated according to the recommended nutritional standards for laying hens in NRC (1994) (Table 1). The experimental pre-feeding period was 1 week, and the formal experimental period was 4 weeks. The GS used in this study is in its sulfate form (purity ≥ 98%), purchased from Wanyuanshan Biotechnology Co., Ltd. (Qingdao, China). It was incorporated into the basal diet using a premixing (stepwise dilution) method to ensure uniform distribution.

### 2.2. Sample Collection

After the initiation of the formal experiment, 3 eggs were randomly selected from each replicate on the 2nd and 4th weekends for egg quality testing. On the 4th weekend, blood samples from laying hens were collected through the inferior vein of the wings. The blood samples were left to settle at 4 °C for serum to precipitate and then centrifuged at 3000 r/min for 10 min using a low-temperature high-speed centrifuge (Eppendorf, Hamburg, Germany) to prepare the serum. We divided the serum into cryovials and stored them in a −80 °C freezer (Haier Biomedical Co., Ltd., Qingdao, China) for an analysis of serum biochemistry and antioxidant indicators. After the experiment, the laying hens were euthanized using rapid bloodletting of the jugular vein, and liver and uterine tissues were quickly removed after dissection. Part of the liver tissue was fixed with 4% paraformaldehyde for histopathological sectioning, while the remaining liver and uterine tissues were rapidly transferred to a −80 °C ultra-low-temperature freezer (Haier Biomedical Co., Ltd., Qingdao, China) after being frozen with liquid nitrogen for subsequent gene expression level detection and transcriptome sequencing analysis.

### 2.3. Determination of Production Performance and Egg Quality

In the second and fourth weeks, we recorded the feed intake and egg production of laying hens on a per replicate basis to calculate the average daily feed intake and laying rate. We used an electronic balance (Sartorius Scientific Instruments Co., Ltd., Beijing, China) to measure the egg weight and calculated the feed-to-egg ratio based on the feed intake, egg weight, and laying rate. All egg quality parameters were measured within 24 h of collection. A multifunctional egg quality tester and eggshell strength tester (Robotmation Co., Ltd., Tokyo, Japan) were utilized to measure the albumen height, Haugh unit, yolk color, and eggshell strength. We then used a spiral micrometer (Yingshi Measurement Technology Co., Ltd., Suzhou, China) to measure the total thickness, membrane thickness, and hard thickness of the eggshell equatorial region.

### 2.4. Pathological Observation of Liver Tissue

After dehydration, transparency, and paraffin embedding, completely fixed liver tissue blocks were sliced into 5 μm thick sections using a paraffin slicer (Shanghai Leica Instrument Co., Ltd., Shanghai, China) and stained with hematoxylin eosin (H&E). We then placed the prepared slices under a microscope (Olympus Corporation, Tokyo, Japan) to observe the morphology and fatty degeneration of the liver tissue and performed a photographic analysis.

### 2.5. Determination of Serum Biochemical Indicators

All serum biochemical indicators were detected using a fully automated biochemical analyzer (Mindray Bio Medical Electronics Co., Ltd., Shenzhen, China). The determination was carried out using commercial reagent kits that were compatible with the instrument. We prepared the reagents and set the analysis parameters (detection wavelength, reaction temperature, reaction time, and sample to reagent volume ratio) according to the reagent kit’s instructions. After thawing and thoroughly mixing the serum samples of laying hens, we began the measurement program as required, and the subsequent steps—such as sample addition, incubation, colorimetry, and result calculation—were automatically completed by the instrument.

### 2.6. Determination of Serum Antioxidant Indicators

The antioxidant enzyme content in the serum was determined using a commercial kit (Jiang Bioengineering Institute, Nanjing, China), and the specific operation strictly followed the instructions.

### 2.7. Transcriptome Sequencing Analysis

We selected 6 liver samples from each of the control groups and the 0.35% GS group for transcriptome sequencing. Total RNA was extracted using TRIzol (Thermo Fisher Scientific, Waltham, MA, USA), and the RNA concentration, purity, and integrity were measured using NanoDrop 2000 (Thermo Fisher Scientific, Waltham, MA, USA) and Agilent 2100 Bioanalyzer (Agilent Technologies, Santa Clara, CA, USA). After the RNA qualification, the data were used to construct a cDNA library and sequenced on the Illumina NovaSeq 6000 platform. After filtering and removing low-quality and adapter sequences from the offline data, HISAT2 was used to align high-quality sequences to the reference genome (*Gallus_gallus*). The gene expression level was expressed as an FPKM value, and a differential expression analysis was performed using DESeq2 software (version 1.40.0). The screening criteria are Log2 (Fold Change) ≥ 1.2 and a *p* value < 0.05. We also performed a KEGG annotation analysis and KEGG pathway enrichment analysis on differentially expressed genes.

### 2.8. Detection of RT-qPCR

The total RNA was isolated from the uterine tissue of laying hens using TRIzol (Thermo Fisher Scientific, Waltham, MA, USA), and the concentration and purity of RNA samples were determined using NanoDrop 2000 (Thermo Fisher Scientific, Waltham, MA, USA). cDNA synthesis was performed using a reverse transcription assay (Abbott Biotech Co., Ltd., Wuhan, China), and an RT-qPCR reaction was performed on the QuantStudio 5 real-time fluorescence quantitative PCR system (Applied Biosystems, Waltham, MA, USA). *β-actin* was used as the internal reference gene, and the relative expression level of the gene was calculated using the 2^−ΔΔCt^ method. The primers for RT-qPCR are shown in Table 2.

### 2.9. Data Processing and Statistical Analysis

All data are presented in the form of mean ± standard deviations. The statistical analysis was conducted on the IBM SPSS Statistics (version 25.0; IBM Corp., Armonk, NY, USA) platform. Inter-group differences were tested using one-way ANOVA. If the analysis of variance revealed significant results, Duncan’s method was further used for multiple comparisons to determine the specific differences between each treatment group. *p* < 0.05 was used as the criterion for determining the significance of differences.

## 3. Results

### 3.1. The Effect of Dietary GS on the Production Performance of Aged Laying Hens

The changes in the production performance of laying hens are shown in Table 3. In the second week, adding 0.15% or 0.35% GS to the diet had no significant effect on the feed intake, laying rate, or feed-to-egg ratio of elderly laying hens (*p* > 0.05). In the fourth week, when the amount of GS added to the daily diet was 0.15%, the hens’ laying rate exhibited an upward trend, while the feed-to-egg ratio demonstrated a downward trend (*p* > 0.05). When 0.35% GS was added, the difference in changes was significant (*p* < 0.05).

### 3.2. The Effect of Dietary GS on the Quality of Eggs in Aged Laying Hens

The changes in egg quality during the second and fourth weeks are shown in Table 4. The addition of GS to the elderly laying hens’ diet had no significant effect on the egg weight, eggshell membrane thickness, albumen height, Haugh unit, or yolk color (*p* > 0.05). In the fourth week, the addition of 0.35% GS significantly improved the eggshell strength, total eggshell thickness, and hard eggshell thickness (*p* < 0.05).

### 3.3. The Effect of Dietary GS on the Liver Tissue Structure of Aged Laying Hens

**Figure 1:**
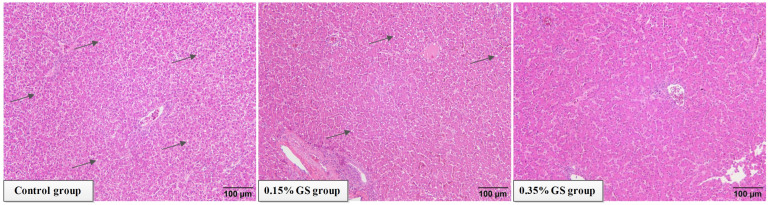
The effect of GS on the liver tissue structure of aged laying hens. Magnified 200 times. The black arrow indicates the site of fat degeneration.

Figure 1 displays the changes in the hens’ liver tissue structure. Diffuse steatosis was observed in the liver of the control group. Images from the histopathological examination revealed fat infiltration in the liver, and vacuoles formed via the accumulation of a large amount of neutral fat in the cytoplasm of parenchymal liver cells were visible in the field of view. In severe cases, vacuolar fusion occurred, pushing the nucleus to one side. Adding GS to the diet may effectively alleviate the symptoms of liver steatosis in elderly laying hens. When 0.35% GS was added, the relief effect was significant, and the vacuolar phenomenon in liver cells was significantly reduced.

### 3.4. The Effect of Dietary GS on Serum Biochemical Indicators of Aged Laying Hens

The changes in serum biochemical indicators of laying hens in the fourth week are shown in Table 5. As the amount of GS added to the diet gradually increases, the levels of ALT, AST, ALP, TG, and TC in the hens’ serum gradually decreases, while the level of ALB gradually increases. When the 0.15% GS is added, there is no significant difference in the changes in the above indicators (*p* > 0.05). When 0.35% GS is added, there is a significant difference in the content changes in AST, ALB, and TG (*p* < 0.05). GS had no significant effect on the levels of TP or GLU in serum (*p* > 0.05).

### 3.5. The Effect of Dietary GS on Serum Antioxidant Indicators in Aged Laying Hens

The changes in serum antioxidant indicators of laying hens in the fourth week are shown in Table 6. With the gradual increase in the GS addition, the MDA content in the serum gradually decreased, while the content of CAT and GSH-Px gradually increased. When the addition was 0.35%, compared with the control group, the difference in the MDA and GSH-Px content was significant (*p* < 0.05). GS had no significant effect on the content of T-SOD in serum (*p* > 0.05).

### 3.6. Overall Changes in Differentially Expressed Genes in the Liver

**Figure 2:**
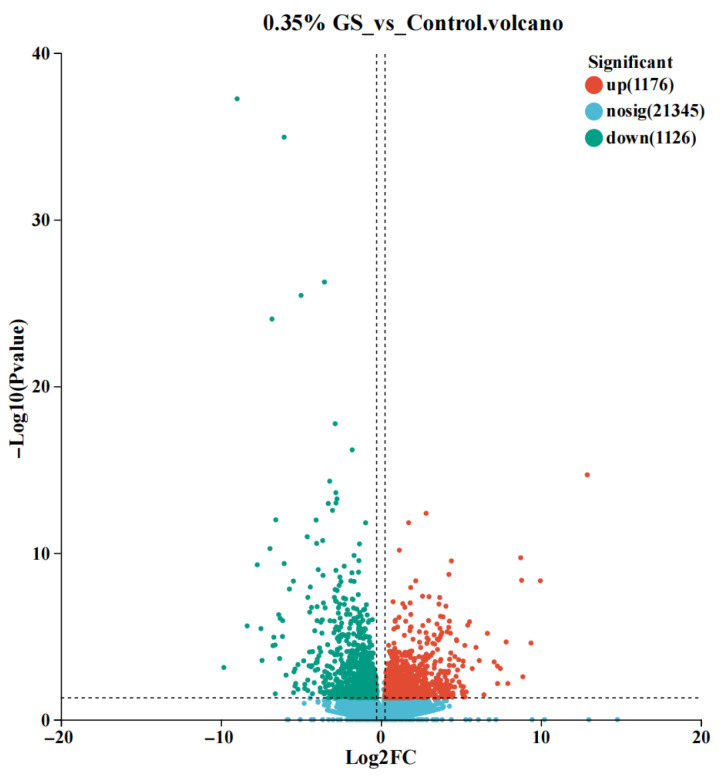
Volcanic diagram of differential gene expression levels in the liver.

The transcriptome changes between the GS treatment group (0.35%) and the control group were compared via the differential expression analysis, and a volcano plot was constructed to visualize the results (as shown in Figure 2). Each point in the graph represents one gene, the horizontal axis represents the fold change in gene expression differences, and the vertical axis represents the negative logarithm of the significance level. The results revealed that there were significant changes in the expression levels of 2302 genes, with 1176 genes exhibiting significant upregulation (Log2 FC > 0) and 1126 genes exhibiting significant downregulation (Log2 FC < 0) (*p* < 0.05).

### 3.7. The Functional Classification of Differentially Expressed Genes in the Liver

**Figure 3:**
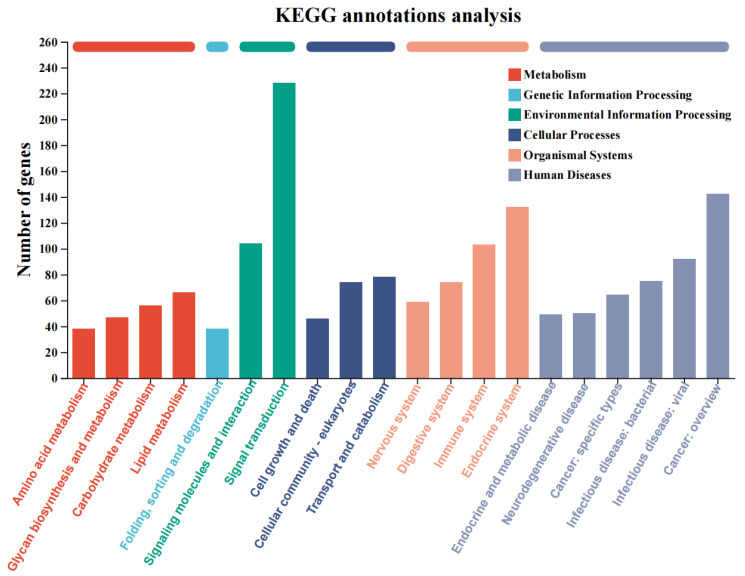
KEGG annotation analysis of differentially expressed genes.

The functional classification of differentially expressed genes was performed via a KEGG annotation analysis, and the results are shown in Figure 3. The functional classifications of differentially expressed genes that are significantly enriched and closely related to liver lipid metabolism regulation and liver health mainly include “lipid metabolism”, “carbohydrate metabolism”, “transport and catabolism”, the “endocrine system”, and the “immune system”.

### 3.8. Functional Enrichment Analysis of Differentially Expressed Genes in the Liver

**Figure 4:**
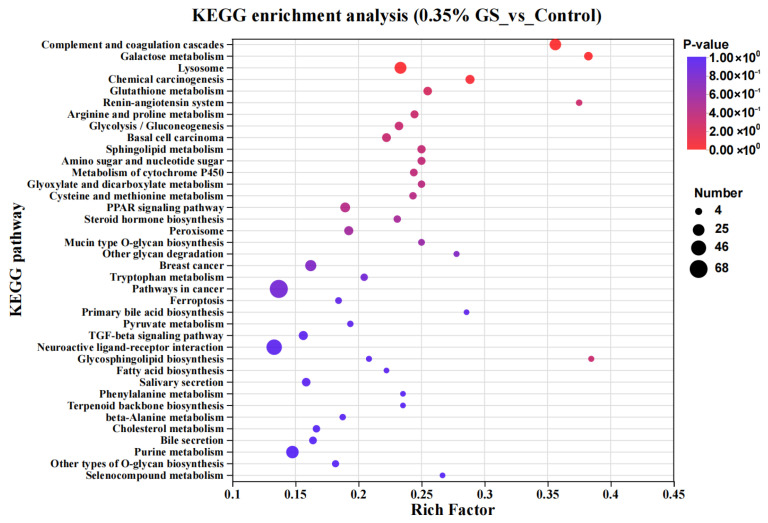
KEGG enrichment analysis of differentially expressed genes.

A further KEGG enrichment analysis was performed on differentially expressed genes, and the results are shown in Figure 4. Compared with the control group, when 0.35% GS was added to the diet, the KEGG pathways closely related to lipid metabolism regulation and liver health were mainly enriched in the liver of laying hens, including “glutathione metabolism”, “sphingolipid metabolism”, the “PPAR signaling pathway”, “steroid hormone biosynthesis”, “peroxisome”, “ferroptosis”, the “TGF-β signaling pathway”, “fatty acid biosynthesis”, “cholesterol metabolism”, and “selenocompound metabolism”.

### 3.9. Changes in Expression Levels of Differentially Expressed Genes in the Liver

In order to more clearly demonstrate the changes in the expression levels of differentially expressed genes in the liver, the differentially expressed genes in the transcriptome were specifically divided into three categories: “lipid metabolism-related genes”, “inflammation-related genes”, and “oxidative damage-related genes” (as shown in Table 7), and the Log2 FC values and significance levels of each gene were also provided. The changes in gene expression levels revealed that in terms of lipid metabolism regulation, the regulatory signals promoting lipid breakdown and inhibiting lipid synthesis were enhanced in the liver of the GS group. The expression levels of fatty acid oxidation- and breakdown-related genes (*PPARA*, *ACOX1*, and *ACOX2*) were significantly upregulated, while the expression levels of fatty acid synthesis-related genes (*PPARG*, *SCD*, and *FASN*) were significantly downregulated (*p* < 0.05). In terms of liver inflammation, the expression levels of multiple inflammation-related genes (*TGFBR2*, *TNF-α*, and *IL-10*) were significantly downregulated in the GS group (*p* < 0.05), and regarding oxidative stress defense, the expression levels of multiple antioxidant-related genes (*GSTA4*, *GSTT1*, *GSS*, *CAT*, *PEX3*, etc.) were significantly upregulated in the GS group (*p* < 0.05).

### 3.10. The Effect of Dietary GS on the Expression Levels of Genes Related to Eggshell Formation in the Uterus of Elderly Laying Hens

**Figure 5:**
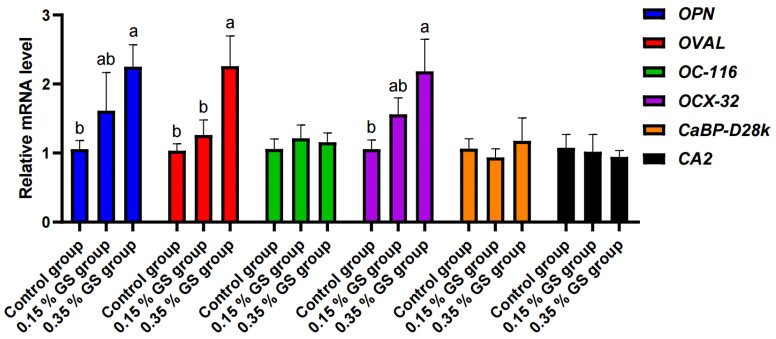
The effect of GS on the expression levels of genes related to eggshell formation in the uterus of elderly laying hens. ^a,b^ Different lowercase letters indicate significant differences between the groups.

The changes in gene expression levels related to eggshell formation in the uterus are shown in Figure 5. GS had no significant effect on the mRNA expression levels of *OC-116*, *CaBP-D28k*, and *CA2* in the uterus (*p* > 0.05). With the gradual increase in the GS addition, the mRNA expression levels of eggshell matrix protein genes—*OPN*, *OVAL*, and *OCX-32*—in the uterus increased, and the difference was significant (*p* < 0.05) with the 0.35% addition.

## 4. Discussion

### 4.1. The Improvement Effect and Mechanism of GS on the Liver Health of Aged Laying Hens

The decline in the liver health of elderly laying hens is one of the key factors that leads to a decrease in their production performance [1,2,12]. This study found that typical diffuse steatosis symptoms appeared in the liver of aged laying hens in the control group, which is consistent with the lipid metabolism imbalance reported in the literature during the late stage of egg laying [12]. This study also found that supplementing 0.35% of GS in the diet not only significantly alleviated symptoms of hepatic steatosis but also significantly reduced levels of AST and TG in the serum, as well as significantly increased levels of ALB. AST is an important indicator that reflects liver cell damage, TG directly reflects the overall level of circulating lipids in laying hens, and ALB is an important marker reflecting liver synthesis function [13,14,15]. These indicators have been synchronously improved, indicating that the GS treatment effectively alleviates liver damage and lipid metabolism abnormalities in aged laying hens, while also restoring the protein synthesis ability of the liver.

The results of the transcriptome sequencing further elucidate the molecular mechanism by which GS improves the liver health of aged laying hens. *PPARA* and *PPARG* are a pair of key nuclear receptors that regulate lipid metabolism in the liver of laying hens: the former dominates the oxidation and degradation of fatty acids, while the latter is mainly responsible for the synthesis and storage of fat [16,17]. GS treatment upregulated the expression of *PPARA* gene and downregulated the expression of *PPARG* in the liver. *ACOX1* and *ACOX2* are downstream target genes of *PPARA*, involved in fatty acid β-oxidation in the liver [18]; *SCD* and *FASN* are downstream genes of *PPARG*, involved in lipid synthesis in the liver [19,20]. GS treatment upregulated the expression of *ACOX1* and *ACOX2*, and downregulated the expression of *SCD* and *FASN* genes. The above results indicate that, GS reshapes the lipid metabolism pattern in the liver of aged laying hens by activating the *PPARA* signaling pathway and inhibiting the *PPARG* signaling pathway, shifting it from a state dominated by synthesis and storage to a state dominated by decomposition and oxidation [16,17]. This is the main reason for the reduction in hepatic steatosis symptoms and the decrease in serum TG and TC levels in the GS group in this study.

In relation to inflammation and oxidative stress damage, the genes *TGFBR2*, *TNF-α*, and *IL-10* are closely related to inflammation in the liver [21,22,23]; And the genes *GSTT1*, *GSTA4*, *CAT*, and *GSS* are closely related to oxidative stress damage in the liver [24,25,26]. The expression levels of multiple inflammation-related genes were significantly downregulated in the liver of the 0.35% GS treatment group, while the expression levels of multiple antioxidant-related genes were significantly upregulated. The results of the KEGG enrichment analysis also revealed that pathways closely related to antioxidant and inflammatory regulation—such as glutathione metabolism, the PPAR signaling pathway, and the TGF-β signaling pathway—were significantly activated or inhibited. Glutathione metabolism is one of the most important intracellular antioxidant defense systems [27]; While the TGF-β signaling pathway is closely related to the development of liver inflammation and liver fibrosis diseases [28,29]. These results indicate that GS not only reduces the metabolic burden of the liver by regulating lipid metabolism but also directly enhances the liver’s antioxidant defense ability and inhibits chronic inflammatory reactions within cells.

At the same time, this study also found that the level of MDA in the serum of aged laying hens in the GS treatment group decreased, while the activities of CAT and GSH-Px increased. MDA is the final product of lipid peroxidation in the body’s cell membrane after being attacked by free radicals, and its content can directly reflect the severity of oxidative damage to the body [30]; GSH-Px is a key intracellular antioxidant defense enzyme responsible for clearing hydrogen peroxide and lipid peroxides [31]. Its activity directly represents the body’s inherent ability to eliminate free radicals and resist oxidative stress [31]; CAT can directly decompose hydrogen peroxide into water and oxygen [32]. The three components together form the core enzyme defense system of the body, eliminating free radicals and resisting oxidative stress [30,31,32]. The changes in the three substances in the serum of the hens in the GS treatment group further validated the antioxidant efficacy of GS from a physiological and biochemical perspective.

Therefore, GS significantly improves the liver health status of elderly laying hens by synergistically regulating lipid metabolism, inhibiting inflammatory reactions, and alleviating oxidative stress through these three core pathways.

### 4.2. The Improvement Effect and Mechanism of GS on the Quality of Aged Eggshells

The decline in eggshell quality is another major problem faced in regard to elderly laying hens [1,4,5]. This study discovered that supplementing 0.35% GS in the diet of elderly laying hens can significantly improve the eggshell strength and egg thickness. Eggshells are formed in the uterus of laying hens, and the key mechanism regulating their formation is the organic matrix secreted by the uterine glands [33]. This organic matrix is rich in various matrix proteins, which can precisely regulate the nucleation, growth, and final assembly of calcium carbonate crystals on eggshells [8,33,34,35]. The results for gene expression levels in the uterus revealed that the GS treatment significantly upregulated the mRNA expression of key eggshell matrix protein genes—*OPN*, *OVAL*, and *OCX-32*—in the uterus. OPN, OVAL, and OCX-32 have been proven to affect calcium carbonate crystals’ growth process and the microstructure in eggshells, thereby enhancing their strength [8,36,37]. Therefore, GS may serve as a precursor for the synthesis of glycosaminoglycans and proteoglycans, promoting the synthesis of these key matrix proteins, optimizing the organic matrix and calcium carbonate crystal structure of eggshells, and ultimately enhancing their strength [8,33,34,35,36,37]. However, the expression levels of genes related to calcium transport and deposition in the uterus (such as *CaBP-D28k* and *CA2*) were not affected, indicating that the main mechanism by which GS enhances eggshell strength is not through the regulation of the uterus’s calcium metabolism process but rather by the enhancement of the organic matrix’s synthesis [38].

### 4.3. The Effect of GS on the Production Performance and Egg Quality of Aged Laying Hens

The production performance and egg quality of laying hens are the core indicators that directly determine the economic benefits of breeding [1]. This study found that in the fourth week of the experiment, the egg production rate of the 0.35% GS group significantly increased, and the feed-to-egg ratio significantly decreased. There may be two reasons for this. Firstly, GS improves laying hens’ lipid metabolism process, enhances liver health, and provides a good physiological basis for the egg laying process [6,39]. Secondly, after enhancing the quality of eggshells, GS reduced the loss during egg production and reduced the degree of ineffective egg production caused by poor eggshell quality, which may also represent one of the important reasons behind the decrease in the feed-to-egg ratio [40]. Wang et al. (2024) reported that dietary GS can significantly increase the protein content and Haugh units of eggs [8]. However, this study found that GS had no significant effect on egg quality indicators other than eggshells, and the results of this study are inconsistent with previous reports. The reason for this may be due to factors such as the breed, age, and breeding cycle length of the laying hens.

After the GS treatment, the hens’ egg production rate, eggshell quality, and liver and body health were synchronously improved, which has important practical value for extending the economic feeding cycle of aged laying hens.

## 5. Conclusions

In summary, adding GS to the diet of elderly laying hens for 4 weeks can significantly improve their production performance, eggshell quality, and liver health, and the antioxidant capacity and health level of the laying hens are also synchronously improved. GS’s mechanism of action involves the simultaneous regulation of the lipid metabolism, inflammatory response, and oxidative stress in the liver and the upregulation of the expression of eggshell matrix protein genes in the uterus. This study provides a theoretical basis for the application of GS as a functional feed additive in the healthy breeding of elderly laying hens.

## Figures and Tables

**Table 1:** Dietary formula and nutritional level.

Feed Ingredients	%	Nutrient Content ^2^	Estimated Value
Corn	62.40	Metabolizable energy	2670 kcal/kg
Soybean meal (43% crude protein)	23.35	Crude protein	16.50%
Rapeseed meal (36% crude protein)	2.40	Calcium	3.60%
Stone powder (Calcium ≥ 36%)	8.20	Total phosphorus	0.53%
Calcium hydrogen phosphate (*p* ≥ 16%)	1.30	Available phosphorus	0.42%
Soybean oil	0.50	Lys	0.82%
Salt	0.30	Met	0.42%
Choline chloride	0.10	Met + Cys	0.70%
L-Lys	0.20		
DL-Met	0.22		
L-Thr	0.03		
Additive premix ^1^	1.00		

^1^ Additive premix can provide per kilogram of diet: Vitamin A, 12,000 IU; Vitamin D_3_ 4000 IU; Vitamin E, 40 mg; Vitamin K_3_, 1.3 mg; Vitamin B_1_, 2.3 mg; Vitamin B_2_, 7 mg; Vitamin B_6_, 6 mg; Vitamin B_12_, 0.015 mg; Niacin, 30 mg; Folic acid, 1.2 mg; Pantothenic acid, 15 mg; Biotin, 0.22 mg; Iron, 60 mg; Copper; 8 mg; Manganese, 90 mg; Zinc, 80 mg; Iodine, 0.8 mg; and Selenium, 0.3 mg. ^2^ Nutritional level is a calculated value.

**Table 2:** Primers for RT-qPCR.

Gene Names ^1^	Accession Number	Primer (5′-3′)	Tm (℃)
*OPN*	NM_204535.5	F: ACAATTACGCGCTAGCAAATCC	58.6
		R: GAGTAATGAGTCTGCTGAAGTG	
*OVAL*	NM_205152.3	F: CAACAATTACGCGCTCAAAGTG	58.6
		R: GTTGGTTGCGATGTGCTTGAT	
*OC-116*	NM_204569.1	F: AACAACAATTACGCGCTATGGC	58.4
		R: TTTTGGCAGAGTCTGTGACAG	
*OCX-32*	NM_204534.5	F: CGCTGCACTACATCAACTCC	58.6
		R: TTGAGCATGCACACACTTCC	
*CaBP-D28k*	NM_205513.2	F: AGGGTGTCAAAATGTGTGCA	58.6
		R: CTCGGTAAAGCTTCCCTCCA	
*CA2*	NM_205317.2	F: ACTGTGGATGGCGTGAAGTA	58.5
		R: TGCAGTGGTGGAGTAGTCAG	
*β-actin*	NM_205518.2	F: AGAGCTATGAACTCCCTGATGG	60.0
		R: TTCGTCATACTCCTGCTTGC	

^1^ *OPN*: Secreted phosphoprotein 1; *OVAL*: Ovalbumin; *OC-116*: Matrix extracellular phosphoglycoprotein; *OCX-32*: Retinoic acid receptor responder 1; *CaBP-D28k*: Calbindin 1; and *CA2*: Carbonic anhydrase 2.

**Table 3:** The effect of GS on the production performance of aged laying hens ^1^.

Items	Control Group	0.15% GS Group	0.35% GS Group	*p* Value
Week 2				
Feed intake (g/bird)	112.83 ± 2.69	114.10 ± 3.24	113.27 ± 2.42	0.659
Laying rate (%)	83.97 ± 3.05	83.42 ± 4.10	83.09 ± 3.97	0.893
Feed-to-egg ratio	2.32 ± 0.24	2.27 ± 0.20	2.35 ± 0.21	0.771
Week 4				
Feed intake (g/bird)	112.91 ± 2.87	113.97 ± 2.42	113.72 ± 3.49	0.760
Laying rate (%)	82.47 ± 4.28 ^b^	84.48 ± 2.67 ^ab^	87.38 ± 2.26 ^a^	0.019
Feed-to-egg ratio	2.35 ± 0.19 ^a^	2.22 ± 0.13 ^ab^	2.12 ± 0.16 ^b^	0.030

^1^ *n* = 8; The results are expressed as mean ± standard deviation; The letters with different superscripts on the same row indicate significant differences.

**Table 4:** The effect of GS on the egg quality of aged laying hens.

Items	Control Group	0.15% GS Group	0.35% GS Group	*p* Value
Week 2				
Egg weight (g)	58.23 ± 4.44	60.65 ± 4.34	58.63 ± 5.78	0.578
Eggshell strength (N)	37.62 ± 4.18	38.38 ± 7.38	38.59 ± 8.11	0.955
Total thickness of eggshell (mm)	0.41 ± 0.04	0.39 ± 0.03	0.40 ± 0.03	0.475
Eggshell membrane thickness (μm)	64.88 ± 15.70	65.75 ± 13.60	62.88 ± 10.06	0.907
Hard eggshell thickness (mm)	0.35 ± 0.03	0.33 ±0.02	0.34 ± 0.03	0.285
Albumen height	7.49 ± 1.55	6.73 ± 1.01	7.35 ± 1.33	0.477
Haugh unit	86.28 ± 9.16	81.26 ± 6.89	84.96 ± 7.76	0.443
Yolk color	12.38 ± 0.92	12.88 ± 1.25	13.50 ± 0.93	0.120
Week 4				
Egg weight (g)	58.59 ± 3.52	61.06 ± 2.38	61.61 ± 3.48	0.152
Eggshell strength (N)	36.27 ± 2.72 ^b^	36.67 ± 2.44 ^b^	41.96 ± 2.94 ^a^	0.001
Total thickness of eggshell (mm)	0.38 ± 0.02 ^b^	0.40 ± 0.01 ^ab^	0.41 ± 0.01 ^a^	0.021
Eggshell membrane thickness (μm)	65.87 ± 9.46	67.50 ± 8.68	68.25 ± 4.77	0.830
Hard eggshell thickness (mm)	0.32 ± 0.02 ^b^	0.33 ± 0.01 ^ab^	0.34 ± 0.01 ^a^	0.012
Albumen height	6.95 ± 1.51	7.35 ± 1.17	7.30 ± 1.39	0.817
Haugh unit	82.90 ± 9.53	84.24 ± 6.25	84.81 ± 7.46	0.884
Yolk color	11.63 ± 1.30	12.63 ± 0.74	12.25 ± 0.89	0.158

*n* = 8; The results are expressed as mean ± standard deviation; The letters with different superscripts on the same row indicate significant differences.

**Table 5:** The effect of GS on serum biochemical indicators of aged laying hens.

Items ^1^	Control Group	0.15% GS Group	0.35% GS Group	*p* Value
ALT (U/L)	2.06 ± 0.71	1.75 ± 0.69	1.73 ± 0.37	0.494
AST (U/L)	256.16 ± 35.96 ^a^	229.55 ± 42.99 ^ab^	193.84 ± 45.06 ^b^	0.023
ALP (U/L)	290.68 ± 76.22	252.31 ± 68.63	234.53 ± 29.34	0.200
TP (g/L)	44.35 ± 2.74	46.48 ± 3.95	44.02 ± 4.15	0.363
ALB (g/L)	20.22 ± 0.98 ^b^	21.40 ± 0.67 ^ab^	21.73 ± 1.60 ^a^	0.040
TG (mmol/L)	11.63 ± 1.61 ^a^	10.14 ± 2.03 ^ab^	8.78 ± 1.73 ^b^	0.016
TC (mmol/L)	2.85 ± 0.56	2.42 ± 0.36	2.30 ± 0.58	0.097
GLU (mmol/L)	18.54 ± 1.13	17.85 ± 0.71	18.11 ± 1.03	0.381

^1^ ALT: Alanine aminotransferase; AST: Aspartate aminotransferase; ALP: Alkaline phosphatase; TP: Total protein; ALB: Albumin; TG: Triglyceride; TC: Total cholesterol; GLU: Glucose. *n* = 8; The results are expressed as mean ± standard deviation; The letters with different superscripts on the same row indicate significant differences.

**Table 6:** The effect of GS on serum antioxidant indicators in aged laying hens.

Items ^1^	Control Group	0.15% GS Group	0.35% GS Group	*p* Value
MDA (nmol/mL)	9.67 ± 0.59 ^a^	8.52 ± 0.45 ^b^	7.36 ± 0.95 ^c^	<0.001
CAT (U/mL)	2.55 ± 0.43	2.79 ± 0.43	2.87 ± 0.21	0.224
GSH-Px (U/mL)	1797.70 ± 80.39 ^b^	1859.20 ± 112.35 ^ab^	1938.51 ± 120.97 ^a^	0.047
T-SOD (U/mL)	84.25 ± 5.88	87.68 ± 4.67	86.94 ±5.48	0.420

^1^ MDA: Malondialdehyde; CAT: Catalase; GSH-Px: Glutathione peroxidase; T-SOD: Total superoxide dismutase. *n* = 8; The results are expressed as mean ± standard deviation; The letters with different superscripts on the same row indicate significant differences.

**Table 7:** Differentially expressed genes within enriched pathways in laying hen livers.

Gene ID	Gene Symbol	Log2 FC	*p* Value	Gene Description
Lipid metabolism-related genes				
374120	*PPARA*	0.86	0.013	Peroxisome proliferator activated receptor alpha
373928	*PPARG*	−2.82	<0.001	Peroxisome proliferator-activated receptor gamma
395706	*SCD*	−1.08	<0.001	Stearoyl-CoA desaturase
424380	*HMGCS2*	2.26	0.046	3-hydroxy-3-methylglutaryl-CoA synthase 2
100858842	*APOA2*	3.24	0.005	Apolipoprotein A-II
426008	*SLC27A1*	−0.99	0.005	Solute carrier family 27 member 1
396061	*FASN*	−1.83	0.015	Fatty acid synthase
427934	*MCAT*	−0.31	0.004	Malonyl-CoA-acyl carrier protein transacylase
424810	*ACSL3*	−0.48	0.004	Acyl-CoA synthetase long chain family member 3
416324	*ACSL6*	−0.69	0.013	Acyl-CoA synthetase long-chain family member 6
417366	*ACOX1*	0.46	<0.001	Acyl-CoA oxidase 1
416068	*ACOX2*	0.77	0.027	Acyl-CoA oxidase 2
Inflammation-related genes				
396399	*TGFBR2*	−0.42	0.012	Transforming growth factor beta receptor 2
374125	*TNF-α*	−0.63	0.027	Lipopolysaccharide induced TNF factor
396165	*BMP4*	−0.97	0.009	Bone morphogenetic protein 4
424325	*ACVR1C*	−0.84	0.029	Activin A receptor type 1C
374249	*GDF5*	0.58	0.002	Growth differentiation factor 5
421461	*LTBP1*	−0.96	0.001	Latent transforming growth factor beta binding protein 1
395282	*ID1*	−1.34	0.040	Inhibitor of DNA binding 1, HLH protein
428264	*IL-10*	−2.59	<0.001	Interleukin 10
Oxidative damage-related genes				
395612	*GSTA4*	0.78	0.048	Glutathione S-transferase alpha 4
396322	*GSTT1*	1.62	0.001	Glutathione S-transferase theta 1
770916	*MGST2*	0.53	0.030	Microsomal glutathione S-transferase 2
396322	*GSR*	0.55	0.007	Glutathione S-transferase theta 1
428135	*GSS*	0.40	0.015	Glutathione synthetase
GGTL3	*GGT7*	−0.62	0.034	Gamma-glutamyltransferase 7
420676	*GGCT*	2.42	<0.001	Gamma-glutamylcyclotransferase
423600	*CAT*	1.59	0.011	Catalase
421669	*PEX3*	0.47	0.022	Peroxisomal biogenesis factor 3
421197	*PEX13*	0.52	0.003	Peroxisomal biogenesis factor 13
415486	*PEX11A*	1.06	0.008	Peroxisomal biogenesis factor 11 alpha
417202	*KYAT1*	0.34	0.038	Kynurenine aminotransferase 1
426612	*SEPHS1*	0.35	0.014	Selenophosphate synthetase 1

## Data Availability

Raw data are retained by the author and may be available upon reasonable request.
